# Ankle Proprioception Deficit Is the Strongest Factor Predicting Balance Impairment in Patients With Chronic Stroke

**DOI:** 10.1016/j.arrct.2021.100165

**Published:** 2021-11-02

**Authors:** Ji-Eun Cho, Hogene Kim

**Affiliations:** a Department of Rehabilitation & Assistive Technology, National Rehabilitation Center, Seoul, South Korea; b Department of Clinical Rehabilitation Research, National Rehabilitation Center, Seoul, South Korea

**Keywords:** Ankle, Balance, Berg balance scale, Proprioception, Rehabilitation, Stroke, BBS, Berg Balance Scale, DF, dorsiflexion, EV, eversion, FM-L, Fugl-Meyer Lower Extremity, INV, inversion, PF, plantar flexion, ROM, range of motion, TUG, Timed Up and Go

## Abstract

**Objective:**

To determine the main factor that predicts balance impairment in patients with chronic stroke.

**Design:**

Cross-sectional study.

**Setting:**

Inpatient rehabilitation hospital and research laboratory.

**Participants:**

A total of 57 patients (42 men, 15 women; mean age 55.7±12.2 years) with chronic symptoms after stroke.

**Interventions:**

Not applicable.

**Main Outcome Measures:**

Primary outcomes were ankle functions, including strength, range of motion, and proprioception, and balance, including Berg Balance Scale score and Timed Up and Go test values. Secondary outcomes included gait kinematics, Fugl-Meyer Scale score, and Fall Efficacy Scale score.

**Results:**

According to the cutoff score <46 on the Berg Balance Scale and the Timed Up and Go test ≥13.5 seconds, 21 patients were classified as having a balance impairment (36.8%). Multivariable logistic regressions showed that ankle proprioception (odds ratio = 3.49; 95% confidence interval, 1.17-10.42) was a significant predictor when coupled with step length (odds ratio = 0.00; 95% confidence interval, 0.00-0.22). A cutoff score of 2.59 for the ankle proprioception value predicts balance impairment in patients with stroke (area under the curve 0.784).

**Conclusion:**

Ankle proprioception can be used to predict balance impairment in patients with stroke.

Balance impairments in patients with stroke hemiparesis frequently cause difficulties in performing activities of daily living.^1^^,^^2^ Better balance is strongly associated with improved performance, including gait function, and negatively associated with fall incidence.^3^ For optimal balance control, the central nervous system integrates visual, vestibular, and proprioceptive information to produce motor commands that coordinate the activation patterns of muscles.^4^ Proprioception plays a crucial role in balance control as one's ability to integrate the sensory signals from various mechanoreceptors to thereby determine body position and movement in space.^5^ However, despite knowledge of the crucial role that proprioception plays in balance control, few studies have focused on this factor in patients with stroke.

Somatosensory impairment is common after stroke; 89% of stroke survivors are affected.^6^ Poststroke proprioception and tactile somatosensation are more impaired in the leg than in the arm, affecting balance and gait.^7^ Somatosensory information from both the joint (proprioception) and skin (tactile) has been demonstrated to be associated with the perception of verticality,^8^ which in turn is related to balance.^9^ This information plays an important role in providing essential feedback about the weight-bearing of the limbs.^10^ Because the ankle-foot complex is the only part of the body contacting the ground, ankle proprioception provides essential information that enables adjustment of ankle position and movement of the upper body to successfully perform balance tasks in patients with stroke.^4^ Although the central processing of proprioceptive signals from the foot-ankle complex is essential for postural and balance control beyond peripheral reflex mechanisms,^11^ their effects on balance is unclear.

Balance is one of the parameters that predict performance in activities of daily living. The Berg Balance Scale (BBS) is one of the most widely used assessment tools of balance and includes multiple items examining different aspects of balance performance.^12^ In stroke populations, BBS cutoff scores have been determined to predict the risk of falls, length of stay and discharge destination in inpatient rehabilitation, and degree of improvement to achieve community walking speed.^12^^,^^13^ Nonetheless, BBS alone did not assess mobility and falls and demonstrated poor prediction of falls after stroke^14^; instead, using a combination of the BBS and the Timed Up and Go (TUG) test, which has been shown to be predictive of balance impairment after stroke, is recommended.^15^ Predicting the risk factors of balance impairment will contribute to our understanding of the parameters that determine balance and can provide the knowledge needed for optimal rehabilitation programs in patients with stroke.

Thus, this study aimed to determine the factors that can predict balance impairment in patients with stroke. In particular, this study focuses on the association between ankle function, including ankle proprioception, and balance ability. We hypothesized that impaired balance is associated with decreased ankle proprioception and related to paretic weight-bearing.

## Methods

### Setting and participants

This cross-sectional study was approved by the institutional review board at the National Rehabilitation Center (No. NRC-2017-04-035), and participants provided written informed consent before study enrollment. Participants were recruited from inpatients at the hospital of the National Rehabilitation Center. The eligibility criteria were as follows: (1) chronic poststroke hemiparesis and (2) independent walking under supervision for gait assessment (functional ambulatory category score >3). Potential participants were excluded if they had complications of orthopedic disorders or severe cognitive impairment (Mini-Mental State Examination score ≤24).

### Outcome measures

**Fig 1:**
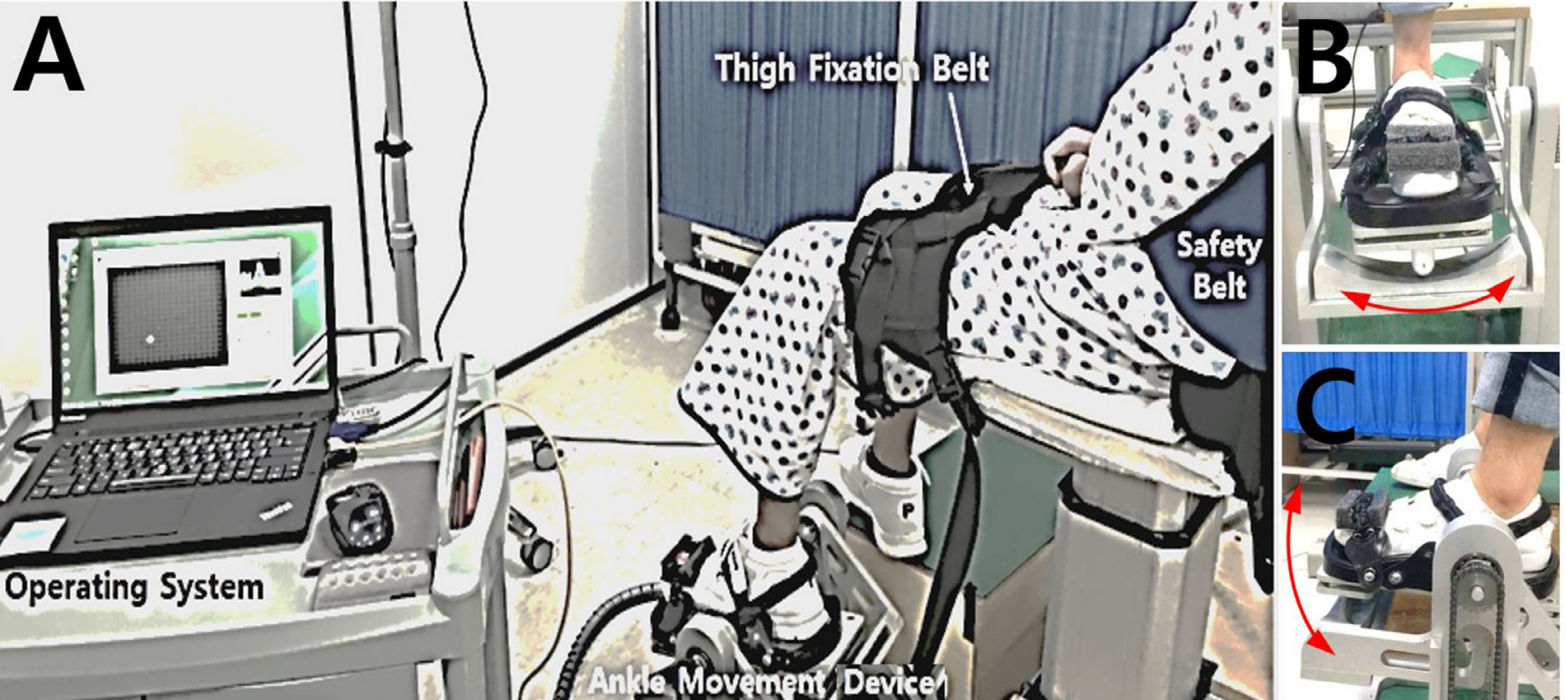
**(A)** Ankle proprioception assessment device. Participants were asked to comfortably sit on a height-adjustable chair with their knees flexed at 90°, to place their paretic foot on the footplate of the ankle movement device, and to place their nonparetic foot on the height-matched footrest. **(B)** The paretic foot was fastened to the force plate in the ankle movement device using 3 length-adjustable straps with boa dials. **(C)** The straps are wide enough and a soft material, sponge, is used between the strap and shoe to avoid pressure concentration.

All outcome measurements were performed by skilled physiotherapists. As an assessment of ankle function, the passive range of motion (ROM) of the paretic ankle was measured using a portable goniometer. The average values of 3 measurements were recorded for the maximum passive ROM of dorsiflexion (DF), plantar flexion (PF), eversion (EV), and inversion (INV). To measure ankle strength, the isometric contraction force of the paretic ankle muscle was measured using a portable manual muscle strength tester. The isometric strength of the ankle dorsiflexor, plantar flexor, invertor, and evertor was measured for 5 seconds and the maximum value was recorded. For the assessment of ankle proprioception, the recognition of a reference position was used in this study. A previous study that measured ankle proprioception with different velocities (0.35°/s, 2°/s, 4°/s, 5°/s, and 10°/s) showed that subjects made larger errors when matching the reference positions at the highest speed.^16^ Furthermore, patients with stroke with damage to the central nervous system had to consider the ankle spasticity that occurs in a speed-dependent manner. Therefore, we measured ankle proprioception at a sufficiently slow speed. In fact, considering the mechanical errors to the set speed in the ankle movement device, the average movement speed of the equipment was measured to 2.14°/s in slow speed mode. The device used for assessing proprioception in this study was developed to provide intensive targeted ankle movement trainings.^17^^,^^18^ This device consists of a foot forceplate with a cradle and supporting frames and its main feature is to reproduce actual biaxial ankle movements applied by the seesaw-type foot cradle that can be simultaneously pivoted along the transverse ankle axis and along a 42°-tilted subtalar axis relative to the foot cradle. In addition, the device can control ankle movements at a desired speed to the preset position and record time and position data. With a custom program via multichannel FPGA controller, the device can control ankle movement at a desired constant speed while minimizing position error.^19^ Participants were asked to comfortably sit on a height-adjustable chair with his/her knees flexed at 90°, to place their paretic foot on the footplate of the ankle movement device, and to place their nonparetic foot on the height-matched footrest (fig 1). Participants wore eye masks and earplugs and were in a sitting position with the other lower limbs fixed to allow only ankle movement. The assessment comprised 2 steps. In the first step, the ankle was moved passively from the initial angle (0°) to randomly assigned 10° target angles (10°, 20°, or 30° for ankle PF and INV; 10° or 20° for ankle DF and EV, according to the normal ROM of the ankle), while asking the participant whether the ankle movement and the direction of movement were perceivable. After staying at the target position for 5 seconds, the ankle was returned to the initial angle. In the second step, the paretic ankle was moved toward the target angle again and the participant was asked to say “stop” when they felt that they had reached the target angle (actual angle). No feedback about results was provided to the participant during the task. The assessment began with a period of familiarization. Three ankle movements were evaluated per direction, and a total of 38 measurements, including dummy trials without movement, were performed. For statistical analyses, proprioception ratios were calculated in relation to angular differences, which means that the difference between the target angle and actual angle^20^ was ascertained, using the following equation:

$\text{Proprioception}\text{ratio}=|\frac{\text{Target}\text{angle}-\text{Actual}\text{angle}}{\text{Target}\text{angle}}|$.

Finally, the proprioception ratio for all 4 directions (DF, PF, INV, and EV) was calculated as the average value of the proprioception ratio, which was measured 3 times for each angle. The larger the proprioception ratio value, the greater the deficit.

For balance assessments, the BBS was used as a clinical test to evaluate static and dynamic balance.^12^ This scale comprises a set of 14 items for the assessment of functional activities in daily life tasks and is considered the criterion standard to test static and dynamic balance abilities. These activities are classified from 0 (*unable*) to 4 (*independent*). The maximum sum of all values is 56 points, and a lower score indicates decreased stability. A cutoff score of <46 in the BBS can be successfully used to identify those who are at risk of falling.^21^ The TUG test was used to assess mobility, as well as both static and dynamic balance.^22^ The test consists of the participant getting up from a chair, walking 3 meters, turning at a designated spot, returning to the seat, and sitting down. The time taken to finish the test is recorded using a stopwatch. The TUG test was performed 3 times with a pause between repetitions and the shortest measured TUG time was selected. A previous study suggested that a TUG time ≥13.5 seconds can be classified as balance impairment.^23^ Because of the acceptable sensitivity (91% and 80%) and specificity (82% and 100%) for the BBS and the TUG test to predict the risk of falling,^21^^,^^23^ a combined cutoff score of BBS<46 and TUG≥13.5 seconds was defined as balance impairment in this study. The motor domain of the Fugl-Meyer Lower Extremity (FM-L) assessment was used to measure motor impairment.^24^ This domain includes measurements of movement, coordination, and reflex action for the hip, knee, and ankle. The FM-L is rated on a 3-point ordinal scale (0 = *cannot be performed*, 1 = *partially performed*, and 2 = *fully performed*). The maximum possible score for the motor domain of the FM-L assessment is 34, corresponding to full sensorimotor recovery. The Korean version of the Fall Efficacy Scale was applied to ascertain the patient's level of confidence in performing activities of daily living.^25^ This self-report questionnaire contains 10 items, each scored on a scale of 0-10, and the total summed score ranges from 0 to 100. A higher score on this scale indicates increased confidence in performing activities of daily living without falling.

For gait assessments, the VICON motion analysis system^a^ was used. The patient's marker attachment was based on the plug-in gait marker set of a previous study.^26^ For the measurement, the patient was allowed to walk a distance of 10 meters 3 times at a comfortable speed. A total of 12 infrared cameras were used to measure the movement of the patient during walking. The mean value of the 3 measurements was used as the analysis data. The first step and the last step were omitted from the analysis to increase the reliability of the measurement data. Gait parameters of the collected data were analyzed using the gait analysis software Visual 3D v6 Professional.^b^ Measured and analyzed gait parameters included the mean walking speed and the step length, time, and width of the paretic side during walking.

### Sample size

The sample size was calculated using the G*Power^c^ program based on a previous multiple regression analysis study. Two predictors in a model explaining the community ambulation in patients with stroke were used in this analysis.^13^ The results showed that 14 patients needed to be included to reject the null hypothesis that the power was 0.95 with an assumption of a statistical significance level at .05.

### Statistical analysis

All statistical analyses were performed using SPSS v21.0 for Windows.^d^ The normal distribution of baseline data was assessed using the Shapiro-Wilk test. The independent *t* test or chi-square test was conducted for comparing balance-impaired and non-balance-impaired groups at baseline (table 1). Forward conditional multivariate logistic regression was used to identify predictors (independent variables) of balance impairment (dependent variable). Spearman's rank-order correlations (Spearman's rho) were used to check for multicollinearity between independent variables; variables with a significant correlation with BBS (*P*>.05) were used in the logistic regression analysis.^13^ Nagelkerke *R*^2^ values were obtained, and each model is presented with *P* values, unstandardized coefficients, and odds ratios with a 95% confidence interval. Receiver operating characteristic curve analysis was also performed to identify factors that predict balance impairment of stroke rehabilitation. The cutoff score and area under the curve that maximized the sensitivity and specificity were calculated for each receiver operating characteristic curve.

**Table 1:** Baseline characteristics of the study population

Characteristics		All(n=57)	Balance-Impaired (n=21)	Non-Balance-Impaired (n=36)	*P* Value
Age (years)		55.7 (12.2)	59.9 (11.4)	53.3 (12.1)	.048*
Sex (male/female)		42/15	16/5	26/10	.748
Weight (kg)		68.8 (9.7)	69.3 (10.4)	68.4 (9.4)	.857
Height (cm)		167.7 (8.9)	168.0 (9.6)	167.5 (8.7)	.740
Time poststroke (months)		12.7 (8.2)	12.3 (9.3)	12.9 (7.6)	.772
Stroke side (R/L)		25/32	11/10	14/22	.331
Modified Ashworth Scale (0/1/1+/2)		3/16/37/1	1/4/16/0	2/12/21/1	.520
K-MMES (score)		27.8 (2.8)	26.5 (2.6)	28.6 (2.6)	<.001*
ROM of the ankle (°)	DF	14.0 (7.2)	11.7 (7.4)	15.39 (6.8)	.060
	PF	133.9 (9.4)	133.2 (7.4)	134.2 (10.4)	.700
	INV	21.6 (5.6)	21.4 (5.5)	21.8 (5.8)	.765
	EV	18.9 (5.3)	18.1 (5.9)	19.3 (5.0)	.429
Strength of the ankle (N)	DF	12.4 (4.7)	10.6 (2.9)	13.5 (5.2)	.052
	PF	14.4 (4.7)	12.9 (2.5)	13.5 (5.5)	.124
	INV	8.4 (2.6)	7.1 (2.4)	9.2 (2.4)	.009*
	EV	7.5 (2.6)	6.7 (2.2)	8.0 (2.8)	.132
Ankle proprioception (score)		1.8 (1.6)	2.4 (1.3)	1.4 (0.9)	.007*
Fugl-Meyer Scale (score)		18.8 (3.3)	16.7 (2.2)	20.0 (3.3)	<.001*
Functional ambulation category (score)		4.8 (0.9)	4.3 (9.6)	5.1 (0.8)	.001*
Berg Balance Scale (score)		46.6 (6.3)	40.5 (6.4)	50.2 (2.0)	.006*
Timed Up and Go (s)		28.6 (15.9)	39.4 (17.7)	22.3 (10.6)	<.001*
Fall Efficacy Scale (score)		49.2 (28.3)	65.5 (23.0)	39.1 (26.8)	.002*
Walking speed (cm/s)		40.5 (24.8)	21.3 (14.1)	51.5 (22.9)	<.001*
Step length (cm)		34.3 (15.0)	20.8 (12.9)	41.1 (11.0)	<.001*
Step time (s)		0.5 (0.1)	0.5 (0.1)	0.5 (0.1)	.604
Step width (cm)		16.3 (7.5)	15.4 (11.1)	16.8 (5.2)	.681

Values represent mean ± SD.

Abbreviations: K-MMES, Korean version of the Mini-Mental State Examination.

⁎ *P*<.05, independent *t* test.

## Results

### Participants

**Fig 2:**
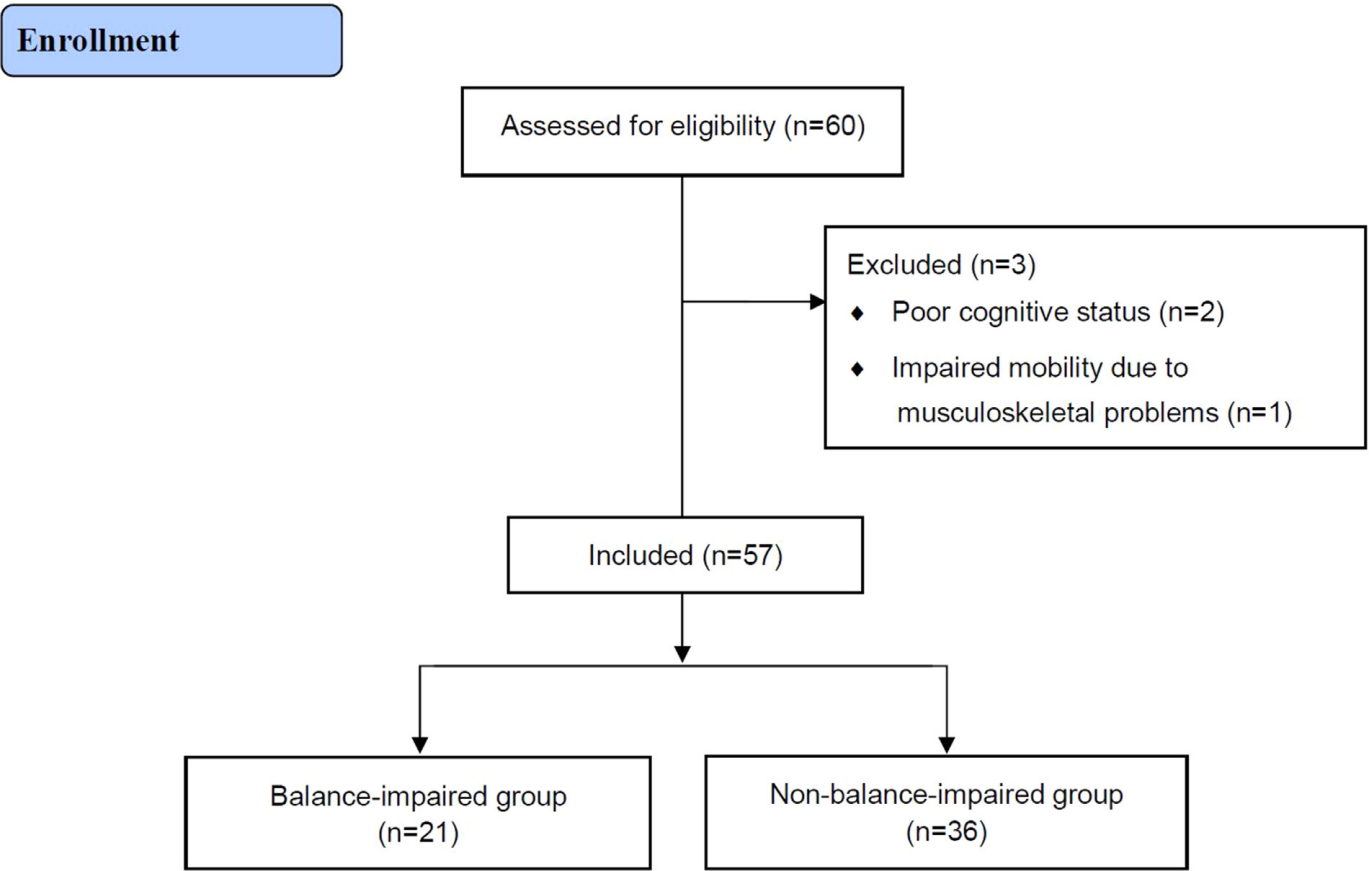
Consolidated Standards for Reporting of Trials (CONSORT) flow diagram.

Of the 60 participants recruited at admission for inpatient rehabilitation, 57 were included in the study for the assessment (fig 2). Of those, 21 were classified as patients with balance impairment (36.8%). The baseline characteristics of the balance-impaired and non-balance-impaired groups are shown in table 1. The parameters age, Korean version of the Mini-Mental State Examination, strength of ankle INV, ankle proprioception, FM-L, functional ambulation category, BBS, TUG, Korean version of the Fall Efficacy Scale, walking speed, and step length were significantly higher in the balance-impaired group compared to the non-balance-impaired group (*P*<.05).

### Correlation with the BBS score

The BBS score was significantly correlated (Spearman's rho>0.3) with the variables age, functional ambulation category, Korean version of the Mini-Mental State Examination, ROM of ankle EV, strength of ankle PF, ankle proprioception, FM-L, TUG, walking speed, step length, and Korean version of the Fall Efficacy Scale (table 2).

**Table 2:** Spearman correlation coefficients between clinical outcome measures and BBS scores (n=57)

Clinical Outcome Measures	Age	FAC	MMES	ROM of Ankle EV	Strength of Ankle PF	Ankle Proprioception	Fugl-Meyer Scale	Timed Up and Go	Walking Speed	Step Length	Fall Efficacy Scale
BBS	−0.323†	0.446‡	0.376†	0.333*	0.346†	−0.401†	0.553‡	−0.621‡	0.732‡	0.701‡	−0.584‡

All values of means and SDs about the variables in table 1 were used in statistical analysis in this table.

Abbreviations: FAC, functional ambulation category; MMES, Mini-Mental State Examination.

Only significant Spearman's correlation coefficients (

⁎ *P*<.05

† *P*<.01

‡ *P*<.001) are reported.

### Predictors of balance impairment

**Fig 3:**
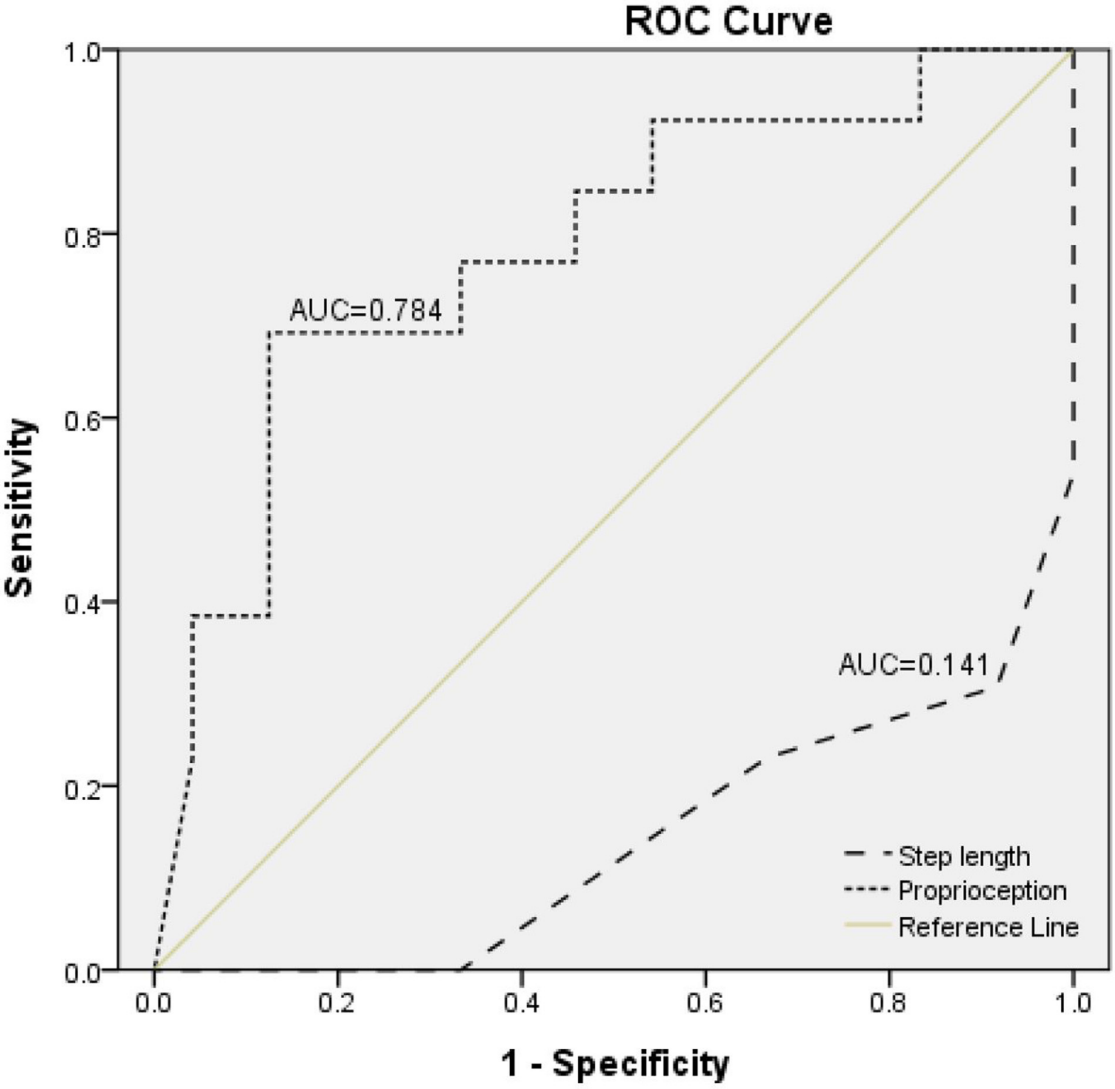
Receiver operating characteristic curves for predicting balance impairment based on ankle proprioception or step length during gait. Abbreviations: AUC, area under the curve; ROC, receiver operating characteristic.

Multivariate logistic analysis showed that ankle proprioception was a predictor of balance impairment in patients with stroke, with an adjusted odds ratio of 3.49 (95% confidence interval 1.17-10.42, *P*<.05; table 3). The step length also remained a significant predictor (*P*<.05) of balance impairment (odds ratio = 0.00; 95% confidence interval, 0.00-0.22). The point on the curve that maximized both sensitivity (0.69) and specificity (0.88) corresponded to a cutoff score of 2.59 on ankle proprioception to predict balance impairment. The optimal cutoff score of a paretic step length value maximizing sensitivity (0.23) and specificity (0.33) was 0.55 meters (fig 3).

**Table 3:** Significant predictors of balance impairment in the multivariate logistic regression models (n=57)

	Initial Model				Final Model			
Variables	*P* Value	B	OR	95% CI	*P* Value	B	OR	95% CI
Fall Efficacy Scale	.149	0.04	1.04	0.99-1.09				
Step length	.026	−17.18	0.00	0.00-0.13	.011	−16.85	0.00	0.00-0.22
Proprioception	.025	1.51	4.53	1.21-16.99	.025	1.25	3.49	1.17-10.42

Nagelkerke *R*^2^: 0.672.

Abbreviations: CI, confidence interval; OR, odds ratio.

## Discussion

This study successfully investigated the correlation between balance impairment and other factors. The most influential factors were ankle proprioception and step length during gait for predicting balance impairment in patients with stroke. The results of this study revealed the importance of the ankle somatosensory system in the balancing strategy of stroke patients.

Balance is closely correlated with the performance of activities of daily living and predicts the level of functional recovery.^27^ The present study showed that the BBS score was significantly correlated with gait-related variables, FM-L, and ankle functions. A review summarizing factors that affect the balance of patients with stroke showed excellent correlations of this score with the Barthel Index, the Postural Assessment Scale, the Functional Reach Test, the Balance subscale of the Fugl-Meyer Scale, the functional independence measure, the Rivermead Mobility Index, and gait speed.^12^ The BBS score also predicted length of stay, discharge destination, motor ability at 180 days after stroke, and disability level at 90 days after a stroke but was not predictive of falls.^12^ Another study revealed that both symmetry of the anteroposterior balance control and weight-bearing during standing were related to swing time and step length symmetry during walking.^28^ This suggests that the balance of patients with stroke reflects the level of physical function and activity including gait performance. Furthermore, physical functions—for example, those of the ankle—that significantly contribute to balance could be included in strategies to improve balance.

Balance control during standing is related to paretic weight-bearing capacity, which requires substantial contribution from ankle proprioception. The results of this study that ankle proprioception is a significant predictor of balance impairment and has 78.4% explanatory power suggest a crucial role of ankle proprioception in balancing. Sensory information from the ankle is associated with the perception of verticality^8^ and contributes to all activities involving weight-bearing. Proprioceptive deficits of the ankle have significant relationships with physical functions; gait parameters such as walking speed, gait symmetry, stride length, and walking endurance; and balance activities in daily living.^29^, ^30^, ^31^, ^32^ However, the influence of sensory information on gait remains controversial. Tactile and proprioception impairments of the paretic lower limb affect walking velocity,^33^ but correlations between the FM-L sensory score and gait velocity have been noted in other studies, although without statistical significance.^34^ Therefore, studies regarding ankle function in patients with stroke have mainly reported that ankle dorsiflexion ROM and plantar flexor strength were effective in the performance of functional activities rather than ankle proprioception.^35^ However, another study suggested that uncertainty regarding the foot position due to impaired ankle proprioception during walking could alter the step length and affect walking speed.^36^ Depending on the lesion location, strokes can damage both motor and sensory neural systems, thereby leading to neurologic impairment.^37^ Furthermore, the planning and execution of voluntary movement require ankle proprioception information on current and predicted body positions; thus, activities such as balancing can be difficult with severely impaired ankle proprioception. Considering that the improvement of ankle motor control requires continuous proprioceptive feedback on muscle lengths and joint angles, it can be inferred that an improvement of ankle proprioception is necessary for recovery of physical function and balancing performance.

The long-term effects of training programs in stroke rehabilitation to improve ankle proprioception have been analyzed. However, evidence for effective proprioception training methods and their effect on functional ability remains unclear. A meta-analysis reported effects of a 2-week proprioception training of the big toe and ankle regarding light touch, postural control, and gait but not proprioception.^38^ In contrast, a recent review reported that improved leg somatosensory function contributes to the improvement of balance but not gait.^39^ The reason for this discrepancy might be that gait after stroke is influenced by various factors, including muscle strength,^32^^,^^34^ spasticity,^36^ somatosensory function,^32^^,^^34^ cognition,^40^ visuospatial perception,^41^ motor function,^34^^,^^40^ and balance.^34^^,^^42^ Moreover, training that targets only ankle proprioception is rare. Nevertheless, the perceptive ability, the main function of ankle proprioception, is essential for postural control and balance.^43^ In addition, individuals with proprioceptive deficits among patients with stroke experience decreased balance confidence, as well as impaired balance and lack of independence in daily living.^29^ Therefore, impaired ankle proprioception is considered an important factor for the recovery of physical function and balance ability in patients with stroke.

### Study limitations

This study has several limitations. First, the sample size was small. Second, most participants were men. Third, individuals who had difficulty walking independently (functional ambulatory category score of >3) were not included because the risk of falling in the gait assessment. Fourth, patients with chronic stroke who had larger variations in stroke onset and functional levels were excluded. Therefore, the findings of this study cannot be generalized to all stroke populations.

## Conclusion

This cross-sectional study demonstrated that ankle proprioception is the strongest predictor of balance impairment for patients with balance impairment after stroke. The results also showed that step length during gait is an additional significant predictor of balance impairment in patients with stroke. These findings are evidence that ankle proprioception should be considered in the lower limb rehabilitation of patients with stroke.

## Suppliers

a. Vicon MX, VICON Motion System Ltd.
b. Visual 3D v6 Professional, C-Motion Inc.
c. G*Power v3.1.9.2; Heinrich Heine University Düsseldorf.
d. SPSS v 21.0 for Windows, SPSS Inc.

## Appendix Supplementary materials

Image, application 1
